# Visual stimuli in the peripersonal space facilitate the spatial prediction of tactile events—A comparison between approach and nearness effects

**DOI:** 10.3389/fnhum.2023.1203100

**Published:** 2023-10-12

**Authors:** Tsukasa Kimura, Jun'ichi Katayama

**Affiliations:** ^1^The Institute of Scientific and Industrial Research, Osaka University, Ibaraki, Japan; ^2^Department of Psychological Science, Kwansei Gakuin University, Nishinomiya, Japan; ^3^Center for Applied Psychological Science (CAPS), Kwansei Gakuin University, Nishinomiya, Japan

**Keywords:** peripersonal space, prediction, multimodal interaction, event-related desynchronization (ERD), event-related spectral perturbation (ERSP), time-frequency analysis, event-related brain potential (ERP)

## Abstract

Previous studies reported that an object in one's peripersonal space (PPS) attracts attention and facilitates subsequent processing of stimuli. Recent studies showed that visual stimuli approaching the body facilitated the spatial prediction of subsequent tactile events, even if these stimuli were task-irrelevant. However, it is unclear whether the approach is important for facilitating this prediction or if the simple existence of stimuli within the PPS is what matters. The present study aimed to scrutinize the predictive function of visuo–tactile interaction in the PPS by examining the effects of visual stimuli approaching the hand and of visual stimuli near the hand. For this purpose, we examined electroencephalograms (EEGs) during a simple reaction time task for tactile stimuli when visual stimuli were presented approaching the hand or were presented near the hand, and we analyzed event-related spectral perturbation (ERSP) as an index of prediction and event-related brain potentials (ERPs) as an index of attention and prediction error. The tactile stimulus was presented to the left (or right) wrist with a high probability (80%) and to the opposite wrist with a low probability (20%). In the approach condition, three visual stimuli were presented approaching the hand to which the high-probability tactile stimulus was presented; in the near condition, three visual stimuli were presented repeatedly near the hand with the high-probability tactile stimulus. Beta-band activity at the C3 and C4 electrodes, around the primary somatosensory area, was suppressed before the onset of the tactile stimulus, and this suppression was larger in the approach condition than in the near condition. The P3 amplitude for high-probability stimuli in the approach condition was larger than that in the near condition. These results revealed that the approach of visual stimuli facilitates spatial prediction and processing of subsequent tactile stimuli compared to situations in which visual stimuli just exist within the PPS. This study indicated that approaching visual stimuli facilitates the prediction of subsequent tactile events, even if they are task-irrelevant.

## 1. Introduction

Primates, including humans, maintain a special representation of the space surrounding the body called peripersonal space (PPS; Brain, 1941; Rizzolatti et al., 1981). Many decades after it was originally conceived of and named, the PPS is still a hot topic in various research areas and has gained increasing attention, especially with systematic reviews in the last 10 years (Cléry et al., 2015; de Vignemont and Iannetti, 2015; Van der Stoep et al., 2015; Hunley and Lourenco, 2018). Two achievements of these reviews are that they have categorized the PPS as having not only a defensive but also a non-defensive function and evaluated the effect of multisensory stimulus processing in the PPS.

The defensive function has been a classical explanation of the importance of the PPS. The approach of a dangerous object (e.g., a knife) could hurt the body; then, it is necessary to predict physical contact before the object touches us and to decide whether to defend ourselves or flee. Therefore, attention to objects in the PPS is important for physical defense, and the PPS is interpreted as a function safety margin (e.g., Cooke and Graziano, 2003; Graziano and Cooke, 2006; Sambo and Iannetti, 2013). In parallel with this interpretation, it is known that even non-dangerous and non-affective stimuli attract attention within the PPS (e.g., Graziano and Gross, 1995; Reed et al., 2006). This non-defensive function has been interpreted as functioning to focus attention on useful objects (e.g., food and tools) within close proximity (e.g., Rizzolatti et al., 1997; Brozzoli et al., 2014). Recent studies have assumed that defense is not the only important function of the PSS but also that non-defense functions and the interaction of each function also matter (e.g., de Vignemont and Iannetti, 2015). The defensive function is important for self-protection, and this is interpreted by an automatic process by which attention is attracted to a specific threat stimulus, whereas the non-defensive function is the active process of allocating attention to useful information around us and preparing for present and future benefits, e.g., perceiving reward prospects in one's surroundings and estimating reward values in changing social contexts (Coello et al., 2018; Gigliotti et al., 2021, 2023). In addition, the benefits from the non-defensive function could be leveraged when the defense is needed (e.g., we can eat the food we get and also throw it at menacing animals as a decoy). Therefore, the importance of the non-defensive function is still drawing attention and discussion in PSS studies on how non-affective information attracts our attention.

When objects are present in the PPS, they are often in the process of approaching the body. If we can see or hear them approaching, this visual and auditory information in the PPS is processed at the bimodal or trimodal neurons, and processing elicits larger neural activity than information presented outside of the PPS (e.g., Rizzolatti et al., 1981; Graziano et al., 1999). Moreover, the combination of these visual (or auditory) stimuli within the PPS and a tactile stimulus facilitates a response to the tactile stimulus (e.g., Gray and Tan, 2002; Makin et al., 2007; Canzoneri et al., 2012). Taking together the approach of an object to the PPS and a multisensory interaction, it is possible that this interaction could be used to predict subsequent contact (tactile event) since the approaching object might subsequently contact the body. It is necessary to keep in mind that such visuo–tactile interaction in the PPS could vary flexibly, e.g., the facilitation effect of multisensory integration in the PPS could be extended to a wider space depending on the meaning of the visual stimuli and the social context (Geers and Coello, 2023). Therefore, the relationship between multisensory integration and interactions with the environment should be considered for each situation. In this study, we focused only on the PPS effect of the approach of non-affective and task-irrelevant visual stimuli and a subsequent tactile stimulus.

Recent studies reported that the approach of non-affective and task-irrelevant visual stimuli facilitates the prediction of a subsequent tactile stimulus. In research by Kimura (2021), visual stimuli were presented, followed by a tactile stimulus to the hand, and these visual stimuli were presented either sequentially approaching the hand or at a fixed distance from the hand (not near each hand). To confirm the event-related desynchronization (ERD) for a subsequent tactile event, event-related spectral perturbation (ERSP) of the electroencephalogram (EEG) was examined. The results showed that ERD in the beta band occurred strongly at the electrodes near the primary somatosensory area, which processes the tactile stimulus when the visual stimuli approached the hand (tactile stimulus presentation site). ERD is one of the analytical indicators of EEG (e.g., Pfurtscheller and Lopes da Silva, 1999; Engel et al., 2001), and ERD of the beta-band activity in the primary somatosensory area reflects the intensity of prediction of subsequent tactile events (e.g., van Ede et al., 2010). Therefore, this study shows that the approach of visual stimuli within the PPS facilitates the spatial prediction of a subsequent tactile stimulus. Moreover, in previous studies examining ERPs, when a tactile stimulus was presented to the opposite hand from the one approached, the amplitude of event-related brain potentials (ERPs) reflecting prediction error increased (Kimura and Katayama, 2015). This phenomenon occurs not only in spatial prediction but also in temporal prediction (Kimura and Katayama, 2017) and in response to the type of stimulus (Kimura and Katayama, 2018).

These results indicate that the approach of visual stimuli in the PPS facilitates the spatial prediction of a subsequent tactile event. However, these studies did not examine the effect of the presentation of visual stimuli immediately near the hand. Therefore, it is unclear whether the approach of stimuli is important for the prediction of subsequent tactile events or if being located within the PPS (i.e., the presentation of visual stimuli near the hand) is sufficient. Considering the safety margin, it is possible to predict subsequent contact at an early stage, before contact, by using the approach of visual stimuli. Furthermore, the facilitation effect of bimodal sensory stimulation on multisensory interactions varies flexibly and could occur even in spaces away from the body; thus, it might be possible to predict the contact of a tactile stimulus from the beginning of the approach of the visual stimuli (e.g., Serino et al., 2015a,b; Bertoni et al., 2021; Geers and Coello, 2023). Considering finite cognitive resources, it is possible to allocate attentional resources just before contact (i.e., near the hand) to predict a tactile stimulus. The present study aimed to scrutinize the predictive function of visuo–tactile interaction in the PPS by examining the effects of visual stimuli approaching the hand and of visual stimuli simply existing near the hand.

We focused on ERDs in the beta-band activity as an index of the spatial prediction caused by the approach of visual stimuli and P3 in ERP as an index of attention to a subsequent tactile stimulus and the prediction error between spatial prediction from this approach and the deviation of the tactile stimulus from this prediction. Even non-dangerous and non-affective stimuli attract attention within the PPS (e.g., Graziano and Gross, 1995; Reed et al., 2006). In addition, the sequential approach of visual stimuli is processed as useful information for the spatial prediction of a subsequent tactile stimulus (e.g., Kimura and Katayama, 2015; Kimura, 2021), and it is possible that this facilitation effect might not be generated by visual stimuli simply existing in close proximity to the body (e.g., Serino et al., 2015a,b; Bertoni et al., 2021; Geers and Coello, 2023). Therefore, we hypothesized that sequentially approaching visual stimuli facilitates the spatial prediction of a subsequent tactile stimulus due to a non-defensive function of the PSS to direct attention to useful information around us. Based on this hypothesis, we predicted that ERDs in the beta band would occur in each condition before the presentation of the tactile stimulus if participants can predict the tactile stimulus (e.g., van Ede et al., 2010) and that the ERD in the approach condition would be larger than that in the near condition if the approach of visual stimuli better facilitates prediction compared with the nearness of the presentation (e.g., Kimura, 2021). P3 reflects attention to the target stimulus (e.g., Duncan-Johnson and Donchin, 1977; Donchin, 1981; Katayama and Polich, 1996a), and its amplitude increases in response to the deviation of a tactile stimulus from spatial prediction due to the approach of visual stimuli (e.g., Kimura and Katayama, 2015). In the present study, participants were asked to perform a simple reaction time task in response to a tactile stimulus, in which it was necessary to allocate attention rapidly to the tactile stimulus. We predicted that the P3 amplitude elicited by the high-probability stimulus in the approach condition would be larger than that elicited by the high-probability stimulus in the near condition if the approach of a visual stimulus induces attention to the direction from which a tactile stimulus approaches, making it easier to predict and quickly allocate attention to the tactile stimulus. In addition, we predicted that the P3 amplitude elicited by the low-probability stimulus in the approach condition would be larger than that elicited by the low-probability stimulus in the near condition if the approach of visual stimuli better facilitates the prediction of a tactile stimulus compared with the near presentation of visual stimuli. In addition, we examined contingent negative variation (CNV; Walter et al., 1964) in ERP before the presentation of tactile stimuli to ensure that the temporal prediction of tactile stimuli did not differ between conditions. CNV reflects the temporal prediction of a subsequent stimulus, including tactile stimuli (Kimura and Katayama, 2015; Kimura, 2021). We predicted that the amplitude of CNV would not differ between conditions if participants could predict the timing of the presentation of the tactile stimulus in both conditions, as was found in previous studies (Kimura and Katayama, 2015; Kimura, 2021).

## 2. Materials and methods

### 2.1. Participants

Twelve undergraduate and graduate students (nine female students and three male students; 18–24 years of age) participated in the experiment, and all were newly recruited for this experiment. All participants were right-handed, had a normal or corrected-to-normal vision, and had tactile sensitivity without hindrance to perform the task, according to their self-report. This experiment was approved by the Kwansei Gakuin University (KGU) Research Ethics Review Board under the KGU Regulations for Research with Human Participants. Written informed consent was obtained from all participants, and their rights as experimental subjects were protected. In this study, this sample size was determined with reference to Kimura and Katayama (2015) by a similar experimental paradigm rather than by prior calculation. Therefore, we conducted a *post-hoc* power analysis to consider the power of the result. The most important analysis of this study was examining the influence of visual stimuli approaching the hand and visual stimuli close to the hand on the prediction of a subsequent tactile stimulus. The analysis of ERD corresponded to this predictive effect; thus, in our results section, we report a *post-hoc* power analysis of the results of ERD analysis.

### 2.2. Stimulus and equipment

#### 2.2.1. Visual and tactile stimuli

**Figure 1:**
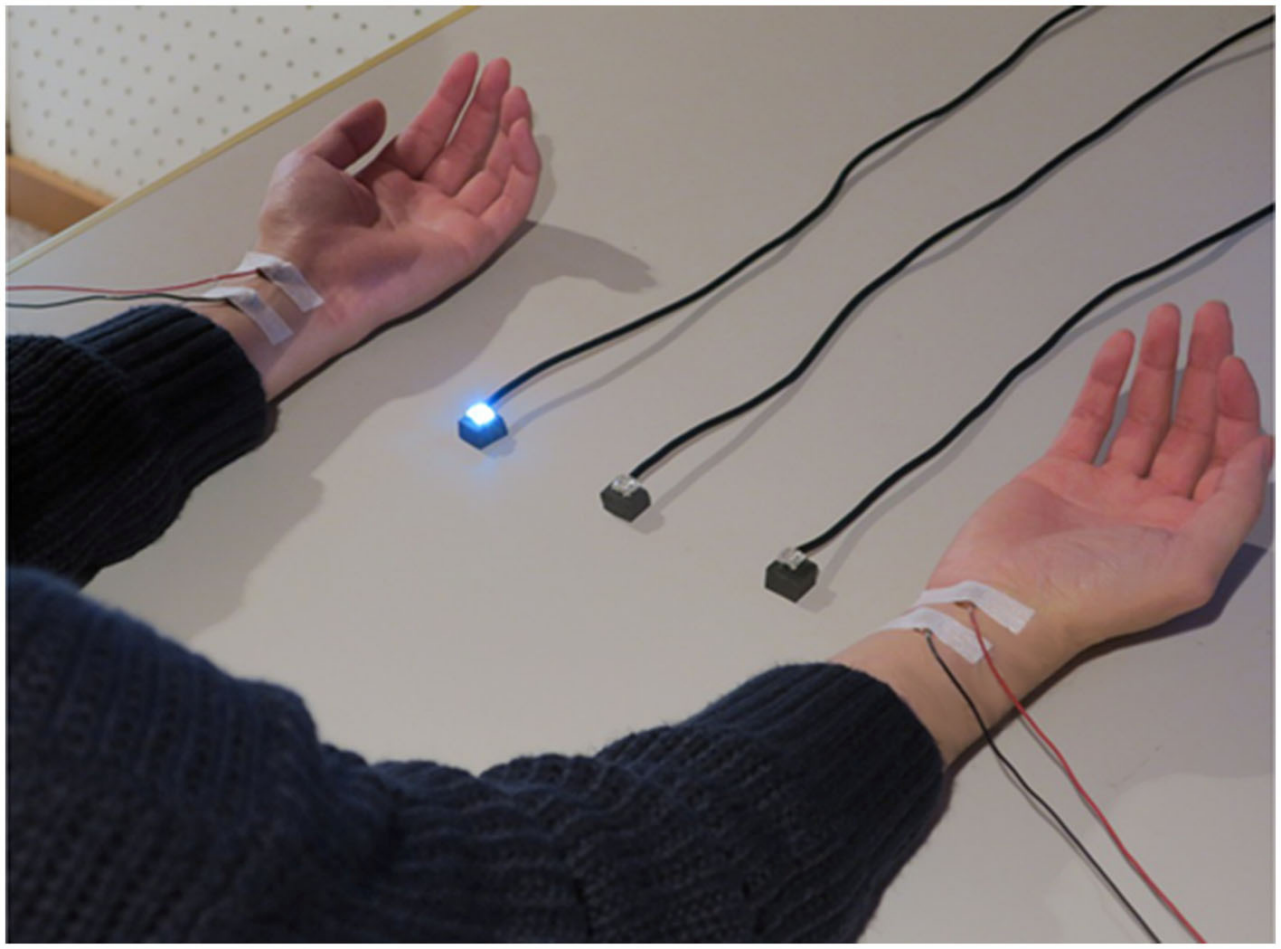
The positions of visual stimuli and tactile stimuli.

Figure 1 shows the positioning of the visual and tactile stimuli. The stimuli were set according to the previous studies (Kimura and Katayama, 2015, 2017, 2018). Participants were seated and put their hands and forearms on a desk in front of them. Their hands were 32.0 cm apart. Visual stimuli were presented by three white light-emitting diodes (LEDs; square with 0.8 cm sides). These were placed between the arms on the desk (at equal distances of 8.0 cm intervals). The intensity and duration of visual stimuli were 25 cd and 200 ms. Somatosensory stimuli were presented by an electrical stimulus generator (Nihon Kohden Corporation, SEN-7203, Japan), electric isolators (Nihon Kohden Corporation, SS-203J, Japan), and Ag/AgCl electrodes (diameter of 1.0 cm) on participants' forearms. The anode electrode was placed on the participants' wrists, and the cathode electrode was 3.0 cm from the anode toward the elbow. The electrical stimulus was a single block pulse with a 0.2-ms duration. The intensities of stimuli were three times as high as the sensory threshold for each participant. This intensity was also used in a previous study of ERP elicited by tactile stimuli (e.g., Kimura and Katayama, 2015), and it never caused pain. The average intensity of the stimuli across all participants was 3.3 mA. The presentation of visual and tactile stimuli was controlled with MATLAB R2010b (MathWorks, Inc.) and Psychtoolbox (Kleiner et al., 2007) installed on a desktop computer (Precision T5500, DELL).

#### 2.2.2. Recording of EEG

EEG data were recorded by NuAmps (Compumedics Neuroscan, USA) and an electrode cap (Easycap GmbH, Germany) using Ag/AgCl electrodes at 30 sites (Fp1, Fp2, F7, F3, Fz, F4, F8, FT7, FC3, FCz, FC4, FT8, T7, C3, Cz, C4, T8, TP7, CP3, CPz, CP4, TP8, P7, P3, Pz, P4, P8, O1, Oz, and O2) according to the modified 10–20 System. In addition, electrodes were also placed on both earlobes (A1 and A2). The reference electrode was on the tip of the nose, and the ground electrode site was AFz. The data from all channels were recorded using SCAN software (Compumedics Neuroscan, USA). The electrode impedances were kept below 5 kΩ. A bandpass filter of 0.1–200 Hz was used for recording. The sampling rate was 1000 Hz.

#### 2.2.3. Procedure

**Figure 2:**
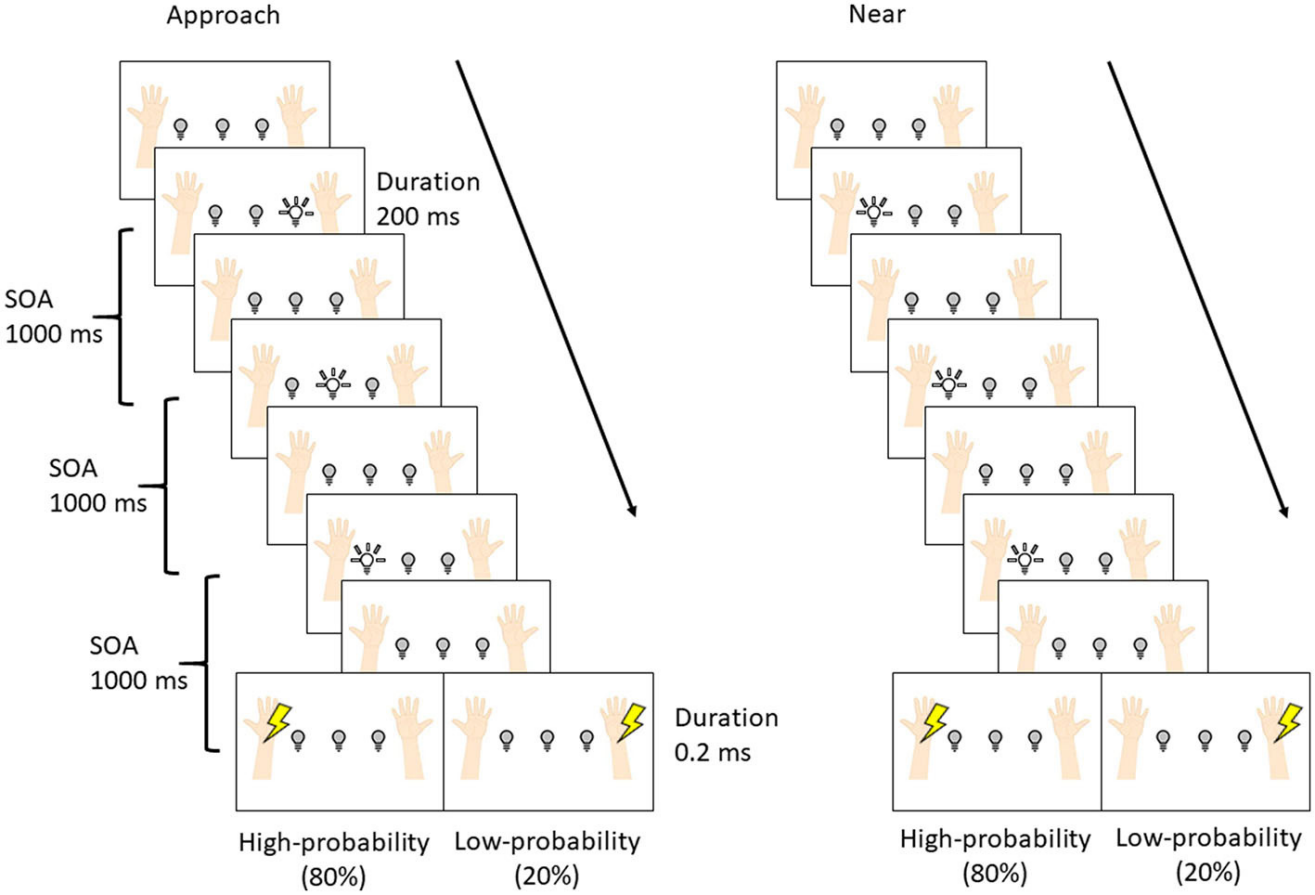
The procedure of the experiment.

Figure 2 shows the experimental procedure. The procedure was set according to the previous studies (Kimura and Katayama, 2015; Kimura, 2021). Each trial was composed of three visual stimuli and one tactile stimulus. The stimulus onset asynchrony (SOA) was set to 1000 ms. The interval between trials was either 1000 or 1200 ms at random with equal probability. Each block was composed of 84 trials [high-probability tactile stimuli: 64 trials; low-probability tactile stimuli: 16 trials; no tactile stimuli (catch trial): 4 trials], which took 7 min. Two blocks were presented for each condition (overall: four blocks per participant). The interval between blocks was 2 min, and after the second block, the participants rested for 10 min and then started the remaining two blocks. The order of conditions was randomized between participants.

The difference between the two conditions was the pattern of visual stimuli, and these patterns were presented in separate blocks. In the approach condition, LEDs flashed sequentially toward the hand where the high-probability tactile stimulus was presented (i.e., if the high-probability tactile stimulus was set at the left wrist, the LEDs flashed sequentially right, center, and left), and the subsequent tactile stimulus was presented to the left (or right) wrist with a high probability (80%) and to the opposite wrist with a low probability (20%). In the near condition, the LED flashed three times near the hand where the high-probability tactile stimulus was presented (i.e., if the high-probability tactile stimulus was set at the left wrist, the left LEDs flashed three times), and the subsequent tactile stimulus was presented to the left (or right) wrist. The participants were required to gaze at the center LED in order to control their eye movements and not to move their eyes and bodies more than necessary in each condition. Moreover, the participants were instructed to respond by pressing a button with the left (or right) foot whenever the tactile stimuli were presented and to not respond when tactile stimuli were not presented (i.e., the catch trials). Half of the participants used the left foot, and the other half used the right foot. Finally, they were told at the start of each block which hand would be presented with the high-/low-probability stimuli.

#### 2.2.4. Data analysis

The mean reaction times (RTs) for the tactile stimuli were calculated. Based on previous studies, trials with an incorrect response or with RTs shorter than 200 ms or longer than 1500 ms were discarded from analysis (Kimura and Katayama, 2015, 2017, 2018). After the rejection, a two-way repeated-measures ANOVA on RTs was conducted with the two conditions (approach and near condition) and two probabilities (high probability and low probability).

The EEG data were analyzed based on the method of Kimura (2021). The EEGLAB toolbox (Delorme and Makeig, 2004) and ERPLAB toolbox (Lopez-Calderon and Luck, 2014) on MATLAB were used for this analysis. Artifacts derived from eye movements and eye blinks were rejected using an automatic EEG artifact detector based on the joint use of spatial and temporal features (ADJUST) of the EEGLAB toolbox (Mognon et al., 2011). In the time-frequency analysis, the EEG data epoch was 1800 ms (including a 900 ms prestimulus of the tactile stimulus). Epochs in which the EEG signal variation exceeded ±100 μV were rejected. After artifact rejection, EEG data were transformed by the Morlet wavelet transformation function applied in a Hanning-tapered window in EEGLAB. The settings were as follows: epoch time limits: −900 to 900 ms, using 400-time points; frequency limits: 8–30 Hz; baseline limits: −900 to −500 ms; wavelet cycles: 3–0.5. The processed data were output from −691.88 to 690.88 ms (400 time points) and from 8 to 30 Hz (22 frequency points). The beta-band (14.29–30 Hz) ERSPs for the time range −300 to 0 ms at the electrodes of C3 and C4 (i.e., the neighboring electrodes for the primary somatosensory area) were averaged in each block. In addition, these electrodes were distinguished by the prediction of a tactile stimulus. C3 (C4) is ipsilateral and C4 (C3) is contralateral when the block with the high-probability tactile stimulus is presented to the left (right) hand. The averaged beta-band ERSP for ipsilateral and contralateral was calculated in each condition. After this processing, the numbers of the remaining trials were 146–160 (0–8.65% rejected) for the approach condition and 149–160 (0–6.87% rejected) for the near condition. To check the ERD, one-sample *t*-tests of beta-band ERSPs were conducted with all combinations between conditions and lateralities (ipsilateral and contralateral). If ERD occurred in all combinations, two-way repeated-measures ANOVAs of ERSPs were conducted with the two conditions and two lateralities.

The ERPs were analyzed based on the method of Kimura and Katayama (2015). The data were digitally low-pass filtered at 30 Hz (6 dB/octave) using an IIR Butterworth analog simulation filter. Artifacts derived from eye movements and eye blinks were rejected using ADJUST. To extract P3, the EEG epoch was set at 1000 ms (including a 200 ms prestimulus of the tactile stimulus). The epoch in which the EEG signal variation exceeded ± 100 μV was excluded from averaging. After artifact rejection, the numbers of remaining trials ranged from 118 to 128 (0–7.81% of trials were rejected) for the high-probability stimuli and 28–32 (0–12.5% rejected) for the low-probability stimuli in the approach condition, and 121–128 (0–5.47% rejected) for the high-probability stimuli and 28–32 (0–12.5% rejected) for the low-probability stimuli in the near condition.

To analyze the P3, the time range of P3 was defined to be 230–290 ms for high-probability stimuli and 260–350 ms for low-probability stimuli. These time ranges were decided by peak latencies of the grand averaged waves for each probability used in the analysis. The mean P3 amplitudes at Pz, where the P3 was elicited at maximum amplitude, were analyzed. Two-way repeated-measures ANOVAs of P3 were conducted with the two conditions and two probabilities. In addition, to investigate CNV, the EEG epoch was set at 1400 ms (the baseline was a – 200 to 0 ms prestimulus of the third visual stimulus). The signal processing and rejection were the same as in the method for P3. After artifact rejection, the numbers of remaining trials were 146–160 (0–8.65% rejected) for the approach condition and 142–160 (0–11.25% rejected) for the near condition. The mean CNV amplitude was obtained from a latency window of 500–1000 ms. The appropriate latency window was defined based on the observation of the resultant ERP waveforms. The mean CNV amplitudes at Cz, where the CNV was elicited at maximum amplitude, were compared between conditions by a paired *t*-test.

The normality of the data for ERD, P3, CNV, and RT was checked by the Shapiro–Wilk test using the R function shapiro.test. These ANOVAs were conducted by applying Greenhouse–Geisser corrections to the degrees of freedom when appropriate (Greenhouse and Geisser, 1959). *Post-hoc* comparisons were made using Shaffer's modified sequentially rejective multiple-test procedure, which extends the Bonferroni *t*-tests in a stepwise fashion (Shaffer, 1986). The effect sizes for ANOVAs were indicated in terms of partial eta squared (${\eta_{p}}^{2}$), and *t*-tests were calculated by computing Cohen's *d*. The significance level was set at a *p*-value of < 0.05 for all statistical analyses.

## 3. Results

### 3.1. Normality test for each data

To confirm the normality of values of ERD, P3, CNV, and RT, Shapiro–Wilk tests were conducted using the R function shapiro.test. The results revealed that these distributions of the data did not significantly differ from a normal distribution (*p*s > 0.05).

### 3.2. *Post-hoc* power analysis

To confirm the statistical power of this study, a *post-hoc* power analysis was conducted using G^*^Power 3.1.9.4 (Faul et al., 2007). The analysis revealed that the statistical power for the result of ERD was 1-β = 0.99 [12 participants, α = 0.05, ${\eta_{p}}^{2}$ = 0.38 (*f* = 0.78)].

### 3.3. Reaction times

**Figure 3:**
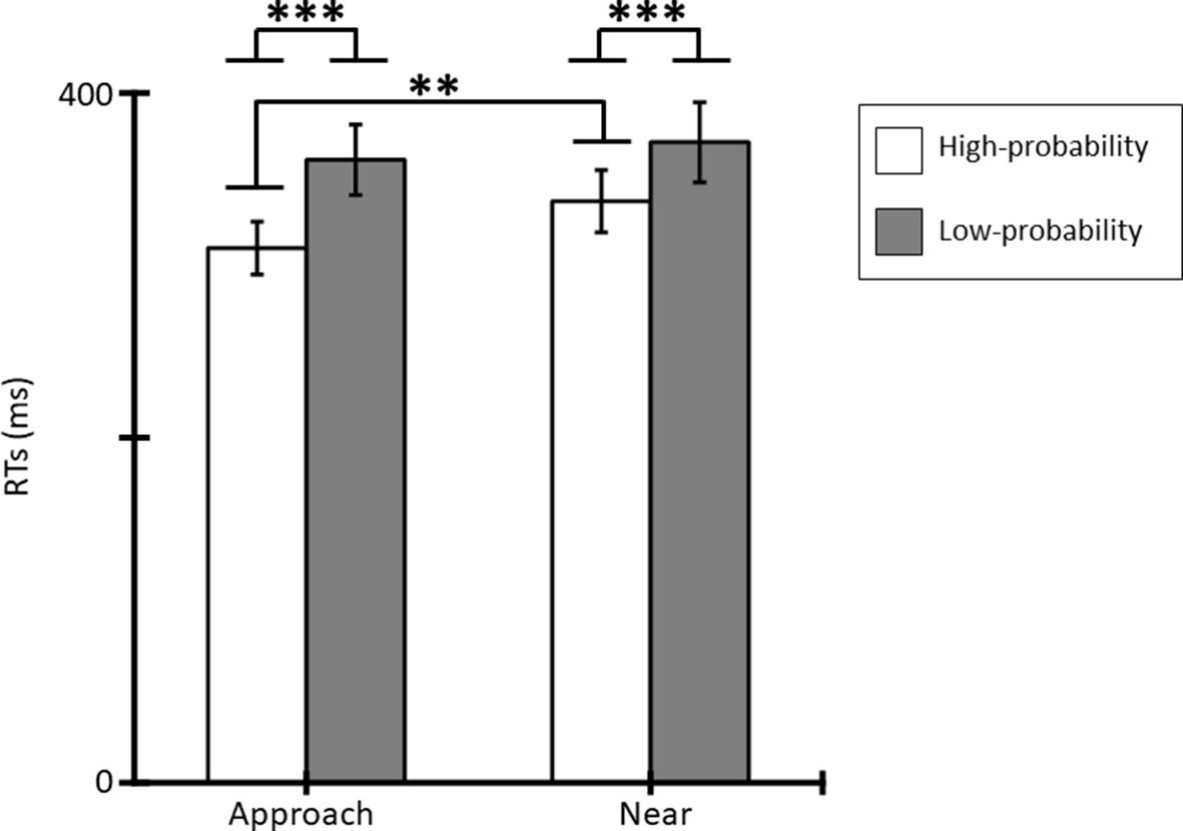
Mean RTs (ms) for tactile stimuli and standard errors (SE) of RTs in each condition (*p < 0.05; **p < 0.01; ***p < 0.001).

Figure 3 shows the mean RTs for the tactile stimuli for each condition. Averaged RTs of all participants were 310 ms (SE = 15.33), 361 ms (SE = 20.44), 337 ms (SE = 18.00), and 372 ms (SE = 23.15) for the approach-high-probability, approach-low-probability, near-high-probability, and near-low-probability stimuli. The results of the ANOVA revealed that the main effect of conditions [*F*_(1, 11)_ = 8.72, *p* = 0.013, ${\eta_{p}}^{2}$ = 0.44] and probabilities [*F*_(1, 11)_ = 27.42, *p* < 0.001, ${\eta_{p}}^{2}$ = 0.71] were significant. In addition, the interaction of conditions and probabilities was significant [*F*_(1, 11)_ = 12.84, *p* = 0.004, ${\eta_{p}}^{2}$ = 0.54]. *Post-hoc* comparisons indicated that the RT in the approach condition was shorter than in the near condition for high-probability stimuli (*p* = 0.002), and low-probability stimuli did not show a significantly different condition effect (*p* = 0.171). In addition, RT to high-probability stimuli was shorter than to low-probability stimuli in each condition (*p*s < 0.001).

### 3.4. Event-related spectral perturbations

**Figure 4:**
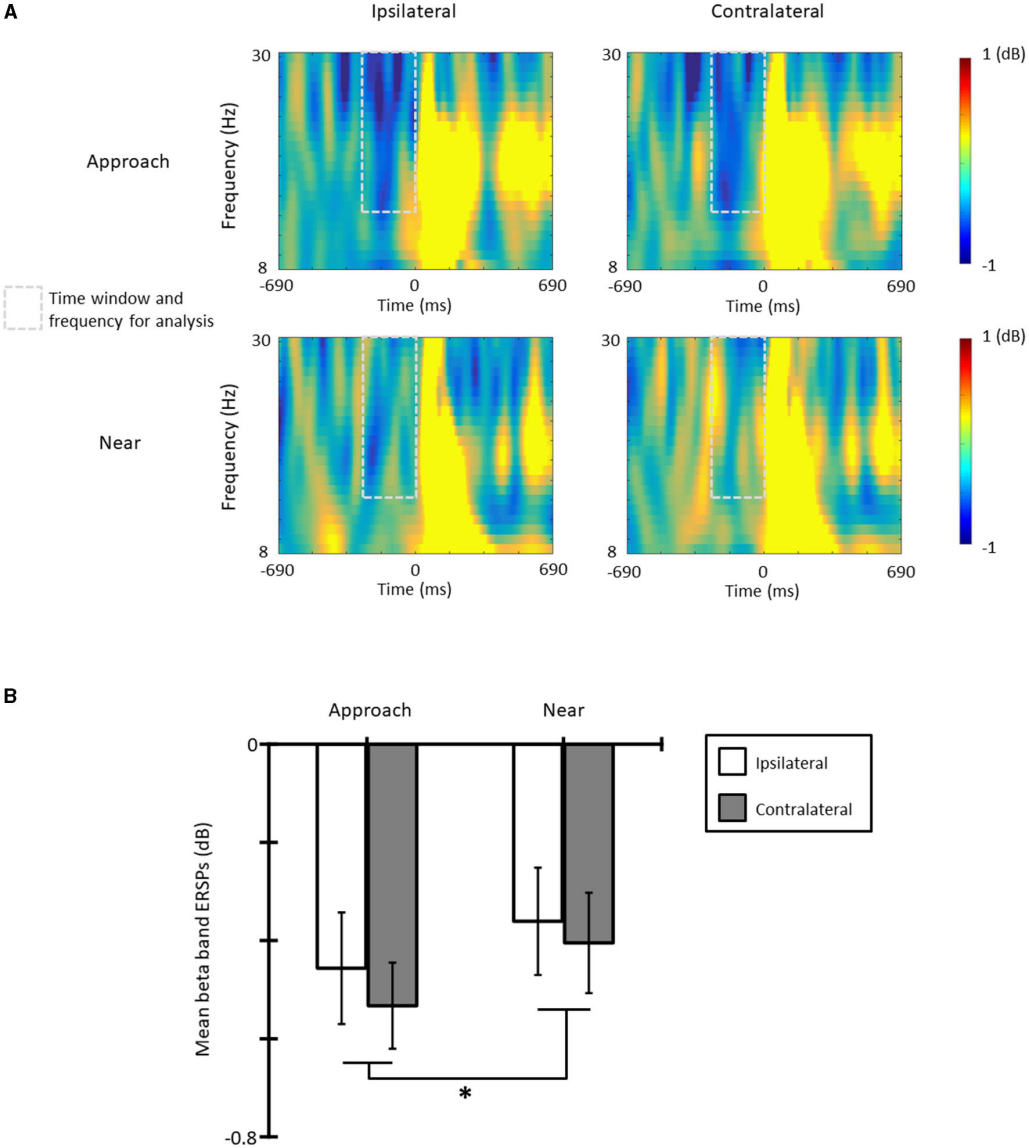
**(A)** The beta band event-related spectral perturbations (ERSPs) in each condition and laterality, and **(B)** the mean beta band ERSPs at the time range of −300 to 0 ms (*p < 0.05; **p < 0.01; ***p < 0.001). The error bars indicate the standard errors (SE).

Figure 4A shows the ERSPs in each condition and each laterality and Figure 4B the averaged beta-band ERSPs at the time range of −300 to 0 ms in all conditions and lateralities. The results of the one-sample *t*-test revealed that the beta-band ERSPs were smaller than zero in all conditions and lateralities [*t*s(11) > 3.30, *p*s < 0.07, *d*s > 1.14]; therefore, ERD occurred in all conditions and lateralities. The results of the ANOVA revealed that the main effect of the condition was significant [*F*_(1, 11)_ = 6.83, *p* = 0.024, ${\eta_{p}}^{2}$ = 0.38] and that the ERD of the approach condition was larger than that of the near condition. The main effect of laterality [*F*_(1, 11)_ = 1.21, *p* = 0.295, ${\eta_{p}}^{2}$ = 0.20] and the interaction [*F*_(1, 11)_ = 0.23, *p* = 0.618, ${\eta_{p}}^{2}$ = 0.02] was not significant.

### 3.5. Event-related brain potentials

**Figure 5:**
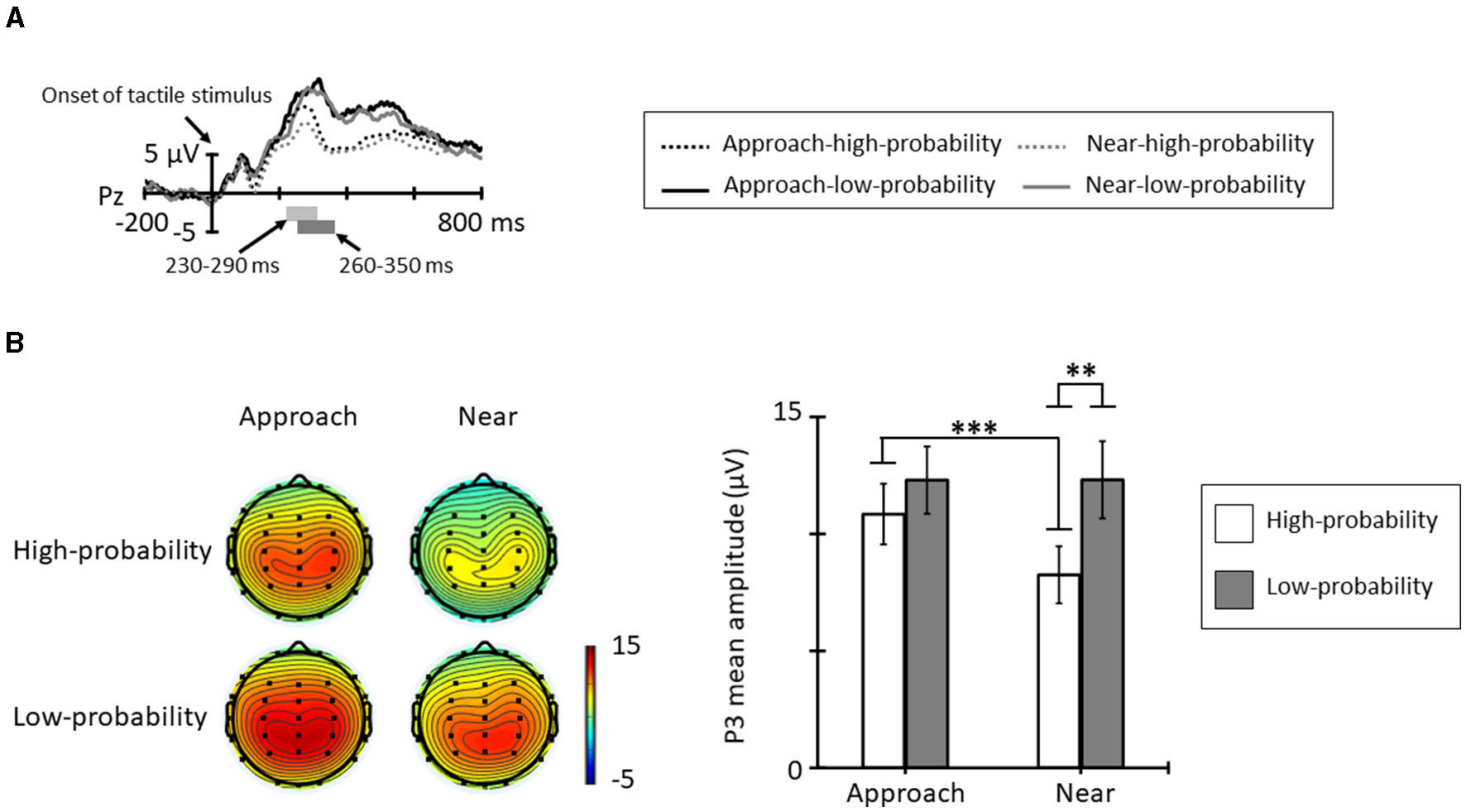
**(A)** Grand average ERP waveforms elicited by tactile stimuli for each condition and probability at Pz. The light gray area denotes the time range of P3 for high-probability stimuli (230–290 ms), and the dark gray area denotes the time range of P3 for low-probability stimuli (260–350 ms). **(B)** The topographic maps and mean amplitudes of P3 for these time ranges (*p < 0.05; **p < 0.01; ***p < 0.001). The error bars indicate the standard errors (SE).

Figure 5A shows the grand averages for ERPs elicited by tactile stimuli in each condition and probability from Pz. The positive deflection in the high-probability stimuli showed peak latency at approximately 260 ms and the positive deflection in the low-probability stimuli showed peak latency at approximately 320 ms. Figure 5B shows the topographic map at the time range and mean amplitude of P3. The ANOVA for the mean amplitude of P3 revealed a significant main effect of probabilities [*F*_(1, 11)_ = 5.18, *p* = 0.044, ${\eta_{p}}^{2}$ = 0.32]; the P3 mean amplitude elicited by the low-probability stimuli was larger than that elicited by the high-probability stimuli. The main effect of conditions was not significant [*F*_(1, 11)_ = 3.99, *p* = 0.071, ${\eta_{p}}^{2}$ = 0.26]. In addition, the interaction of conditions and probabilities was significant [*F*_(1, 11)_ = 8.73, *p* = 0.013, ${\eta_{p}}^{2}$ = 0.44]. *Post-hoc* comparisons indicated that the P3 mean amplitude in the approach condition was larger than in the near condition for high-probability stimuli (*p* < 0.001), and low-probability stimuli did not show a significantly different condition effect (*p* = 0.992). In addition, the probability effect of the P3 mean amplitude shown in the near condition for low-probability stimuli was larger than that elicited by the high-probability stimuli (*p* = 0.005), and this effect did not show in the approach condition (*p* = 0.321).

**Figure 6:**
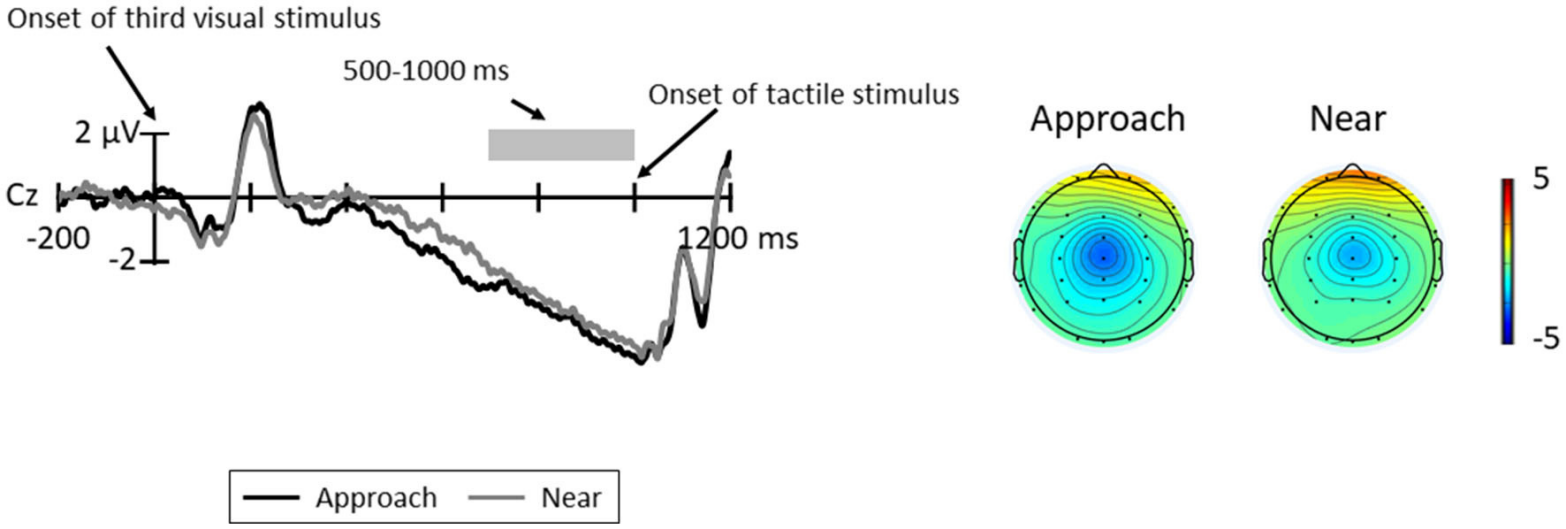
Grand average ERP waveforms between the third visual stimuli and tactile stimuli for each condition at Cz; the gray area indicates the time range for CNV (500–1000 ms). The topographic maps indicate the mean amplitudes of CNV for these time ranges.

Figure 6 shows the grand average CNV elicited in all trials at Cz, where the CNV was elicited at maximum amplitude and the topographic map at the time range of CNV (5000–1000 ms). The results of the paired *t*-test revealed no significant difference between conditions [*t*(11) = 0.32, *p* = 0.754, *d* = 0.12].

## 4. Discussion

This study aimed to scrutinize the predictive function of visuo–tactile interaction in the PSS by examining the effect of visual stimuli approaching the hand and visual stimuli close to the hand. For this purpose, ERDs, ERPs, and RTs were compared between the approach condition and the near condition.

RTs to high-probability stimuli were shorter than those to low-probability stimuli in each condition, and RTs to high-probability stimuli in the approach condition were shorter than those to the high-probability stimuli in the near condition. In addition, the amplitude of CNV did not differ between the conditions. These results suggest that the participants could predict the timing of the presentation of the tactile stimulus in both conditions and that the spatial prediction of the high-probability tactile stimulus is facilitated in the approach condition.

The beta-band ERSPs were suppressed approximately 300 ms before the presentation of the tactile stimulus in all conditions and lateralities. More importantly, the beta-band ERD of the approach condition was larger than that of the near condition. The beta-band ERD before the presentation of a tactile stimulus reflects the intensity of the prediction of a subsequent tactile stimulus (van Ede et al., 2010), and the approach of visual stimuli facilitates this prediction (Kimura, 2021). The only difference between conditions was the method of presentation of visual stimuli. Therefore, this result suggests that the approach of visual stimuli is more important for the prediction of a subsequent tactile stimulus compared with the presentation of visual stimuli near the hand. The tactile stimulus in this study did not cause pain, and the visual stimuli were merely flashes of LED lights; thus, it is possible that these results were caused by a non-defensive function of the PPS. Visual stimuli approaching the body offer useful information for the spatial prediction of subsequent tactile stimuli (e.g., Kimura and Katayama, 2015; Kimura, 2021), and the non-defensive function of the PPS allocate more attention to useful information in the PPS (e.g., Rizzolatti et al., 1997; Brozzoli et al., 2014). Moreover, previous studies reported that an object approaching the PPS activates bimodal and trimodal neurons and then promotes stimulus processing (e.g., Rizzolatti et al., 1981; Graziano et al., 1999). The distance at which these neuronal activities (and the resulting facilitation effect of stimulus processing within the PPS) occur varies flexibly (e.g., Serino et al., 2015a,b; Bertoni et al., 2021; Geers and Coello, 2023). Taken together, the results of this study suggest that visual stimuli approaching the hand might increase the activity of these neurons, facilitating the spatial prediction of a subsequent tactile stimulus within the PPS, even when the stimuli are non-threatening, while the mere presence of visual stimuli in close proximity to the hand might not.

The P3 amplitude elicited by the high-probability stimuli in the approach condition was larger than that in the near condition. P3 reflects attention to a stimulus, and this attention is allocated based on the importance and meaning of the stimulus (e.g., Duncan-Johnson and Donchin, 1977; Donchin, 1981; Katayama and Polich, 1996b). In this study, participants performed a simple reaction time task in response to a tactile stimulus and were required to allocate attention quickly to the stimuli presented. Therefore, it is considered that approaching visual stimuli attracts attention to the space that is approached and that this attention is allocated to a high-probability tactile stimulus that is presented there. As a result, P3 amplitude was increased for high-probability stimuli in the approach condition. Moreover, the P3 amplitude elicited by the low-probability stimuli did not differ between conditions. In the near condition, this amplitude was larger than that elicited by the high-probability stimuli. The results of RT, ERD, and P3 elicited by the high-probability stimuli suggest that the approach condition attracted attention to the presentation location of the high-probability tactile stimulus and facilitated the prediction of the stimulus, whereas the degree of spatial deviance of the low-probability tactile stimulus did not differ between conditions because the distance from one wrist to the other wrist at which the high-probability stimulus was presented is comparable. Therefore, it is possible that spatial prediction errors did not differ between conditions. Moreover, this result may also have been caused by the ceiling effect. In previous studies using similar conditions as the approach condition, the P3 amplitude elicited by low-probability stimuli was comparable to the results in this study (e.g., Kimura and Katayama, 2015). Therefore, the P3 amplitude elicited by low-probability stimuli may not increase further in experiments using this paradigm.

In summary, the present study indicates that the approach of visual stimuli facilitates the spatial prediction and processing of a subsequent tactile stimulus compared to situations in which visual stimuli only exist, without moving, within the PPS. In this study, visual stimuli were task-irrelevant, and the subsequent tactile stimulus also produced no pain; thus, the approach of the visual stimuli within the PPS is considered to relate to an automatic predictive function that facilitates the prediction of subsequent tactile events, even if the visual stimuli are non-affective.

Finally, it is necessary to consider, in a future study, whether this effect influences other predictions for visuo-tactile processing. The approach of visual stimuli facilitates not only spatial prediction but also temporal prediction manipulated by the presentation timing of a tactile stimulus (Kimura and Katayama, 2017) and the prediction of stimulus type manipulated by tactile stimulus features (Kimura and Katayama, 2018). However, it is unclear whether this effect is caused by the approach of visual stimuli or the presentation of visual stimuli within the PPS. Therefore, whether this effect affects the overall prediction for visuo-tactile processing should be investigated in future research.

## 5. Conclusion

The results of this study revealed that the approach of visual stimuli better facilitates spatial prediction and processing of a subsequent tactile stimulus compared to situations in which visual stimuli just exist within the PPS. This study extended our understanding of attentional processing within the PPS and indicated that visual stimuli within the PPS might be related to an automatic predictive function that facilitates the prediction of subsequent tactile events, rather than only having a defensive function.

## Data availability statement

The raw data supporting the conclusions of this article will be made available by the authors, without undue reservation.

## Ethics statement

The study involving human participants was reviewed and approved by the Kwansei Gakuin University (KGU) Research Ethics Review Board under the KGU Regulations for Research with Human Participants. The patients/participants provided their written informed consent to participate in this study.

## Author contributions

TK and JK contributed to the conception and design of the study. TK contributed to the data acquisition and wrote the first draft of the manuscript. Both authors contributed to the statistical analysis, revising and reading the manuscript, and finally approved the submitted version.

## References

[B1] Bertoni T. Magosso E. Serino A. (2021). From statistical regularities in multisensory inputs to peripersonal space representation and body ownership: insights from a neural network model. Eur. J. Neurosci. 53, 611–636. 10.1111/ejn.14981 32965729 PMC7894138

[B2] Brain W. R. (1941). Visual disorientation with special reference to lesions of the right cerebral hemisphere. Brain J. Neurol. 64, 244–272. 10.1093/brain/64.4.244

[B3] Brozzoli C. Ehrsson H. H. Farnè A. (2014). Multisensory representation of the space near the hand: from perception to action and interindividual interactions. The Neurosci. 20, 122–135. 10.1177/1073858413511153 24334708

[B4] Canzoneri E. Magosso E. Serino A. (2012). Dynamic sounds capture the boundaries of peripersonal space representation in humans. PloS ONE 7, e44306. 10.1371/journal.pone.0044306 23028516 PMC3460958

[B5] Cléry J. Guipponi O. Wardak C. Ben Hamed S. (2015). Neuronal bases of peripersonal and extrapersonal spaces, their plasticity and their dynamics: knowns and unknowns. Neuropsychologia 70, 313–326. 10.1016/j.neuropsychologia.2014.10.022 25447371

[B6] Coello Y. Quesque F. Gigliotti M. F. Ott L. Bruyelle J. L. (2018). Idiosyncratic representation of peripersonal space depends on the success of one's own motor actions, but also the successful actions of others!. PloS ONE 13, e0196874. 10.1371/journal.pone.0196874 29771982 PMC5957367

[B7] Cooke D. F. Graziano M. S. (2003). Defensive movements evoked by air puff in monkeys. J. Neurophysiol. 90, 3317–3329. 10.1152/jn.00513.2003 12801896

[B8] de Vignemont D. Iannetti F. (2015). How many peripersonal spaces?. Neuropsychologia 70, 327–334. 10.1016/j.neuropsychologia.2014.11.018 25448854

[B9] Delorme A. Makeig S. (2004). EEGLAB: an open source toolbox for analysis of single-trial EEG dynamics including independent component analysis. J. Neurosci. Method. 134, 9–21. 10.1016/j.jneumeth.2003.10.009 15102499

[B10] Donchin E. (1981). Surprise! … surprise? Psychophysiology 18, 493–513. 10.1111/j.1469-8986.1981.tb01815.x 7280146

[B11] Duncan-Johnson C. C. Donchin E. (1977). On quantifying surprise: the variation of event-related potentials with subjective probability. Psychophysiology 14, 456–467. 10.1111/j.1469-8986.1977.tb01312.x 905483

[B12] Engel A. K. Fries P. Singer W. (2001). Dynamic predictions: oscillations and synchrony in top-down processing. Nat. Rev. Neurosci. 2, 704–716. 10.1038/35094565 11584308

[B13] Faul F. Erdfelder E. Lang A. G. Buchner A. (2007). G^*^ Power 3: a flexible statistical power analysis program for the social, behavioral, and biomedical sciences. Behav. Res. Methods 39, 175–191. 10.3758/BF03193146 17695343

[B14] Geers L. Coello Y. (2023). The relationship between action, social and multisensory spaces. Sci. Rep. 13, 202. 10.1038/s41598-023-27514-6 36604525 PMC9814785

[B15] Gigliotti M. F. Bartolo A. Coello Y. (2023). Paying attention to the outcome of others' actions has dissociated effects on observer's peripersonal space representation and exploitation. Sci. Rep. 13, 10178. 10.1038/s41598-023-37189-8 37349516 PMC10287734

[B16] Gigliotti M. F. Soares Coelho P. Coutinho J. Coello Y. (2021). Peripersonal space in social context is modulated by action reward, but differently in males and females. Psychol. Res. 85, 181–194. 10.1007/s00426-019-01242-x 31493049

[B17] Gray R. Tan H. Z. (2002). Dynamic and predictive links between touch and vision. Exp. Brain Res. 145, 50–55. 10.1007/s00221-002-1085-x 12070744

[B18] Graziano M. S. Cooke D. F. (2006). Parieto-frontal interactions, personal space, and defensive behavior. Neuropsychologia 44, 845–859. 10.1016/j.neuropsychologia.2005.09.009 16277998

[B19] Graziano M. S. Reiss L. A. Gross C. G. (1999). A neuronal representation of the location of nearby sounds. Nature 397, 428–430. 10.1038/17115 9989407

[B20] Graziano M. S. A. Gross C. G. (1995). “The representation of extrapersonal space: a possible role for bimodal, visual-tactile neurons,” in The Cognitive Neurosciences, ed Gazzaniga M. S. (London: The MIT Press), 1021–1034.

[B21] Greenhouse S. W. Geisser S. (1959). On methods in the analysis of profile data. Psychometrika 24, 95–112. 10.1007/BF02289823

[B22] Hunley S. B. Lourenco S. F. (2018). What is peripersonal space? An examination of unresolved empirical issues and emerging findings. Wiley interdisciplinary reviews. Cognit. Sci. 9, e1472. 10.1002/wcs.1472 29985555

[B23] Katayama J. Polich J. (1996a). P300 from one-, two-, and three-stimulus auditory paradigms. Int. J. Psychophysiol. 23, 33–40. 10.1016/0167-8760(96)00030-X 8880364

[B24] Katayama J. Polich J. (1996b). P300, probability, and the three-tone paradigm. Electroencephal. Clin. Neurophysiol. 100, 555–562. 10.1016/S0168-5597(96)95171-0 8980420

[B25] Kimura T. (2021). Approach of visual stimuli facilitates the prediction of tactile events and suppresses beta band oscillations around the primary somatosensory area. Neuroreport 32, 631–635. 10.1097/WNR.0000000000001643 33843822 PMC8048733

[B26] Kimura T. Katayama J. (2015). Approach of visual stimuli modulates spatial expectations for subsequent somatosensory stimuli. Int. J. Psychophysiol. 96, 176–182. 10.1016/j.ijpsycho.2015.04.002 25889695

[B27] Kimura T. Katayama J. (2017). Visual stimuli approaching toward the body influence temporal expectations about subsequent somatosensory stimuli. Brain Res. 1664, 95–101. 10.1016/j.brainres.2017.03.030 28389236

[B28] Kimura T. Katayama J. (2018). The approach of visual stimuli influences expectations about stimulus types for subsequent somatosensory stimuli. Exp. Brain Res. 236, 1563–1571. 10.1007/s00221-018-5244-0 29572648

[B29] Kleiner M. Brainard D. Pelli D. (2007). What's new in psychtoolbox-3? Perception 36, 1. 10.1177/03010066070360S101

[B30] Lopez-Calderon J. Luck S. J. (2014). ERPLAB: an open-source toolbox for the analysis of event-related potentials. Front. Hum. Neurosci. 8, 213. 10.3389/fnhum.2014.00213 24782741 PMC3995046

[B31] Makin T. R. Holmes N. P. Zohary E. (2007). Is that near my hand? Multisensory representation of peripersonal space in human intraparietal sulcus. J. Neurosci. 27, 731–740. 10.1523/JNEUROSCI.3653-06.2007 17251412 PMC6672897

[B32] Mognon A. Jovicich J. Bruzzone L. Buiatti M. (2011). ADJUST: An automatic EEG artifact detector based on the joint use of spatial and temporal features. Psychophysiology 48, 229–240. 10.1111/j.1469-8986.2010.01061.x 20636297

[B33] Pfurtscheller G. Lopes da Silva F. H. (1999). Event-related EEG/MEG synchronization and desynchronization: basic principles. Clin. Neurophysiol. 110, 1842–1857. 10.1016/S1388-2457(99)00141-8 10576479

[B34] Reed C. L. Grubb J. D. Steele C. (2006). Hands up: attentional prioritization of space near the hand. *J. Exp. Psychol*. Hum. Percep. Perf. 32, 166–177. 10.1037/0096-1523.32.1.166 16478334

[B35] Rizzolatti G. Fadiga L. Fogassi L. Gallese V. (1997). The space around us. Science 277, 190–191. 10.1126/science.277.5323.190 9235632

[B36] Rizzolatti G. Scandolara C. Matelli M. Gentilucci M. (1981). Afferent properties of periarcuate neurons in macaque monkeys. II visual responses. Behav. Brain Res. 2, 147–163. 10.1016/0166-4328(81)90053-X 7248055

[B37] Sambo C. F. Iannetti G. D. (2013). Better safe than sorry? The safety margin surrounding the body is increased by anxiety. J. Neurosci. 33, 14225–14230. 10.1523/JNEUROSCI.0706-13.2013 23986256 PMC6618504

[B38] Serino A. Canzoneri E. Marzolla M. di Pellegrino G. Magosso E. (2015a). Extending peripersonal space representation without tool-use: evidence from a combined behavioral-computational approach. Front. Behav. Neurosci. 9, 4. 10.3389/fnbeh.2015.00004 25698947 PMC4313698

[B39] Serino A. Noel J. P. Galli G. Canzoneri E. Marmaroli P. Lissek H.. (2015b). Body part-centered and full body-centered peripersonal space representations. Sci. Rep. 5, 18603. 10.1038/srep18603 26690698 PMC4686995

[B40] Shaffer J. P. (1986). Modified sequentially rejective multiple test procedures. J. Am. Stat. Assoc. 81, 826–831. 10.1080/01621459.1986.1047834119051220

[B41] Van der Stoep N. Nijboer T. C. Van der Stigchel S. Spence C. (2015). Multisensory interactions in the depth plane in front and rear space: a review. Neuropsychologia 70, 335–349. 10.1016/j.neuropsychologia.2014.12.007 25498407

[B42] van Ede F. Jensen O. Maris E. (2010). Tactile expectation modulates pre-stimulus beta-band oscillations in human sensorimotor cortex. NeuroImage 51, 867–876. 10.1016/j.neuroimage.2010.02.053 20188186

[B43] Walter W. G. Cooper R. Aldridge V. J. McCallum W. C. Winter A. L. (1964). Contingent negative variation: an electric sign of sensorimotor association and expectancy in the human brain. Nature 203, 380–384. 10.1038/203380a0 14197376

